# Involvement of Renin-Angiotensin System in Retinopathy of Prematurity - A Possible Target for Therapeutic Intervention

**DOI:** 10.1371/journal.pone.0168809

**Published:** 2016-12-29

**Authors:** Madhu Nath, Parijat Chandra, Nabanita Halder, Baskar Singh, Ashok Kumar Deorari, Atul Kumar, Rajvardhan Azad, Thirumurthy Velpandian

**Affiliations:** 1 Dept. Of Ophthalmology, Dr. Rajendra Prasad Center for Ophthalmic Sciences, All India Institute of Medical sciences, New Delhi, India; 2 Dept. Of Ocular pharmacology, Dr. Rajendra Prasad Center for Ophthalmic Sciences, All India Institute of Medical sciences, New Delhi, India; 3 Dept. Of Biophysics, All India Institute of Medical sciences, New Delhi, India; 4 Dept. Of NICU, Pediatrics, All India Institute of Medical sciences, New Delhi, India; Monash University, Melbourne, Australia, AUSTRALIA

## Abstract

**Objective:**

Examining the Retinal Renin Angiotensin System (RRAS) in the ROP neonates and analyzing the possibility of modulating the RRAS to prevent the progression in Oxygen Induced Retinopathy (OIR) model.

**Method:**

Vitreous of ROP patients (n = 44, median age 5.5 months) was quantified for RRAS components, VEGF, HIF-1α and compared with age matched control. The involvement of RRAS in ROP was tested in the rat model of OIR and compared with normoxia. Expressions of RAS components, VEGF and HIF-1α in retina were analyzed using qPCR and retinal structure and function was also analyzed. Effect of Angiotensin Converting Enzyme Inhibitor (ACEI) and Angiotensin Receptor Blocker (ARB) was evaluated and compared with Bevacizumab which served as a positive control. Drug penetration into retina was confirmed by liquid chromatography coupled ESI-tandem mass spectroscopy (LC-MS/MS).

**Results:**

Multifold increase in the expression of RAS components in human vitreous and rat retina showed their involvement in ROP. ERG & fundus studies in OIR revealed the altered function of retina and were successfully prevented by ARB (telmisartan), ACEI (lisinopril) and bevacizumab. Retinal analysis revealed the presence of ACEI and ARB in their therapeutic levels.

**Conclusion:**

This study for the first time demonstrates the upregulated level of RAS components in human ROP vitreous and further that the pharmacological intervention in RRAS can functionally and structurally preserve retina against the progression of ROP in the OIR model.

## Introduction

Renin Angiotensin System (RAS) is the potent mechanism involved in the homeostatic control of arterial pressure, tissue perfusion, and extracellular volume. The key initiator molecule of this system-renin is secreted from kidney, cleaves the circulating angiotensiongen produced from liver into decapeptide angiotensin I. Further, in the presence of angiotensin converting enzyme which is majorly produced in lungs, the decapeptide angiotensin I is converted into octapeptide angiotensin II, which is a potent vasoconstrictor and helps in maintaining the body fluid homeostasis via blood pressure regulation.

In 1996, Wagner et al., demonstrated the mRNA expression of renin, angiotensinogen and angiotensin converting enzyme in the human eye and suggested the evidence of intraocular renin angiotensin system [1]. Many other studies also reported expression and localization of renin angiotensin system in animal and human ocular system, and hence hypothesized the existence of ocular renin angiotensin system [2,3,4,5].

In many clinical trials it has been seen that angiotensin converting enzyme inhibitor lisinopril was able to reduce the amount of retinopathy in diabetic patients [6]. Thus role of RRAS has been extensively studied for the diabetic retinopathy [7,8]. Retinopathy of prematurity is also one of the pathological conditions where developing retina of premature infant is vulnerable for hyper activation of retinal renin angiotensin system. In 2005, Sarlos et al. reported that RAS is involved in the developing retinal vasculature of rat pups though very few studies have documented the role of renin angiotensin system for the pathological basis of retinopathy of prematurity (ROP) [9,10,11].

ROP is the blinding disease of childhood which does not have any definite pharmacological treatment as of now. The role of RRAS is a very poorly understood domain. Systemically, this system is involved in maintenance of vascular tone and overexpression of this system causes pathological conditions. If the RRAS works through the same mechanism, then modulation of this system can be beneficial in controlling the progression of retinopathy feature of this disease and therefore could be very helpful for many premature infants who are prone to develop this disease. Therefore, this study was carried out to evaluate the involvement of ocular renin angiotensin system in the pathogenesis and to analyze the possibility of modulating RRAS to prevent or halt the progression of retinopathy.

This study has been conducted in two parts in which the involvement of various angiogenic factors along with the hyper-activation of RRAS pathway was studied. First part of the study involved the quantification of RAS components along with Vascular Endothelial Growth Factor (VEGF) and Hypoxia Inducible Factor-1α (HIF-1α) in the vitreous of the babies suffering from ROP who were selected to undergo vitrectomy surgery and compared with age matched controls. The second part of the study evaluated the involvement of RRAS in the simulated retinopathy model in rat pups by quantifying the retinal expression of various factors using qPCR. In the same model ACEI and ARB were evaluated and compared with anti-angiogenic compounds and retina was assessed by using fundus imaging and electroretinography (ERG). Studies were also conducted to quantify the systemic and intraocular penetration of intradermally injected test compounds to confirm their adequacy for the pharmacological action.

## Materials and Methods

### Human Study

#### Renin angiotensin component in vitreous humour of ROP patients

The study was conducted in accordance with ARVO guidelines and the protocol (IESC/T-374/04.10.2013) was approved by the Institute Ethics Committee (All India Institute of Medical Sciences, New Delhi, India). The written informed consent was obtained from all participants legal guardians. The vitreous humour was collected from 44 ROP patients undergoing vitrectomy following ROP surgery. Vitreous from 12 controls were obtained who were undergoing congenital cataract surgery. The vitreous humour was collected in microtubes and stored in -80°C till further analysis. An approximate volume of 800–1000 μL was obtained after vitrectomy sample. Protein and collagen concentration were performed to determine and normalize the consistency of samples.

#### Total protein concentration

Total protein concentration was measured in the vitreous by the Bradford method. Sample and standards were added in the 96 well plate and plate was shaked orbitally for 5 sec. Absorbance was taken at 595 nm and concentration was measured in mg/ml.

#### Total collagen assay

Total collagen assay was done using Sircol collagen kit (Biocolor,Inc). Collagen standards were prepared in triplicate from 0–15 μg/ml. Manufacturer instructions were followed to carry out the experiment. The collagen concentration was expressed as μg/mg of total protein.

#### Enzyme-linked Immunosorbant Assay (ELISA) for renin, angiotensinogen, angiotensin converting enzyme, angiotensin II, Vascular Endothelial Growth Factor and Hypoxia Inducible Factor-1 Alpha

Direct ELISA kits for renin, angiotensinogen, angiotensin converting enzyme, angiotensin II, VEGF & HIF-1 alpha were obtained from Elabsciences Biotechnology Co.,Ltd (China). The assay was performed according to manufacturer’s instructions. Briefly 100 μl of standard and samples were incubated in ELISA plates precoated with the specific antibody and incubated at 37°C for 90 minutes. Fluid was aspirated and 100 μl biotinylated detection antibody was added to wells and incubated for 1 hour at 37°C. Fluid was aspirated and plate washed for 3 times with wash buffer. 100 μl Avidin-Horseradish Peroxidases (HRP) was added to wells and incubated for 1 hour at 37°C. Fluid aspirated and plate washed for 5 times with wash buffer. 90 μl of substrate reagent was added to wells and incubated for 15 minutes at 37°C 50 μl of stop solution was added to wells to stop the reaction. Absorbance was taken at 450 nm.

### Animal Study

#### Exposure protocol

This study was conducted in accordance with ARVO guidelines; the power of animals for the sample size was calculated according to ARVO guidelines and protocol (689/IAEC/12) was approved by institute animal ethics committee (All India Institute of Medical Sciences, New Delhi, India). The OIR model was developed as described by Lonchampt et al., (2001), with the modification of species of animal [10]. The wistar rat mother and pups were kept in humidified oxygen chamber at the oxygen saturation of 75% ± 2% from postnatal day 7 to postnatal day 12 (hyperoxia), then returned to normal room air (Normoxia) from postnatal day 12 to postnatal day 17, inducing relative hypoxic condition. Unexposed animals were kept in room air. At the end of day 12, pups were randomized into different treatment groups (n = 9) i.e. ACEI-lisinopril (0.49mg/kg), ARB-telmisartan (7 mg/kg), antibody against VEGF-bevacizumab (100mg/kg), normoxia and disease control group. All drug doses were calculated from pediatric dosage of these drugs and were given intradermally in two divided doses of 48 hours apart to rule out mortality. The animal’s physical condition was monitored on twice a daily basis. No mortality observed in any of the experimental group. At Day 17 rat pups (n = 9) were anesthetized using ketamine (50mg/kg), subjected for fundus imaging and assessment of ERG. 72 hours after last dosing animals were sacrificed using excess of anesthesia, blood was collected from cardiac puncture, retina and vitreous were carefully removed under operating microscope and fresh tissues were processed for gene expression studies of RAS components. Plasma, vitreous and retina samples were frozen at -80°C for the subsequent quantification of lisinopril and telmisartan using LC-MS/MS.

#### Fluorescein Isothiocynate (FITC) conjugated dextran retinal flat mount

For assessment of establishment of OIR model FITC conjugated dextran method was used to label the vasculature of retina (n = 4) in order to assess the retinal ischemia using method described by Lonchampt et al 2001 [10].

#### Imaging protocol: Functional analysis of retina using Electro Retinogram (ERG)

Rat pups were dark adapted overnight for the ERG evaluation (n = 9). The rat pups were anesthetized using ketamine (50 mg/kg). Eyes of the rat pups were dilated using tropicamide 0.8% and phenylephrine 5%. The corneal electrode was in touch with the centre of the cornea. The ground electrode was connected to the tail of the animal. The reference electrode was placed on the forehead. The ERG responses were obtained through ERG attachment of MICRON III rodent imaging system using labscribe software (Phoenix laboratory. USA) and were performed according to ISCEV guidelines. The retina was stimulated using white light of 30 cds/m^2^ intensity using 6 msec flash duration, 25 averages mean were taken for obtaining single ERG response from rat pup retina. The ‘a’ and ‘b’ wave amplitude and latency were measured in all groups, using inbuilt algorithm of labscribe software. The retinal responses were filtered with a 30-Hz cutoff frequency and low-pass filtered at 300 Hz. The oscillatory potentials filtered, were analyzed for the latency and amplitude of each wavelet using inbuilt software provide by phoenix laboratory.

#### Analysis of retina using fundus imaging

At day 17, following electroretinogram study, retina of rat pups (n = 9) was focused with the objective lens and posterior pole images of both eyes were captured using streampix software using MICRON III rodent imaging system (phoenix lab, USA). The retinal images were then processed into grey version for appropriate tracing of vessels and the tortousity index of arteries and veins were calculated using Image J software (available at http://rsb.info.nih.gov/nih-image; developed by Wayne Rasband, National Institutes of Health, Bethesda, MD) with the standard taken as 100 pixels/mm length as has been described previously by Liu et al. The straight length of the vessel, and the actual length were measured by using the above software and their ratio was calculated to get tortousity index of arteries and veins using the same protocol of Liu et al (2006) [12].

#### Gene expression studies on RAS components and angiogenesis factors

On day 17, rat pups were sacrificed and retina was extracted for gene expression analysis (n = 9). Renin, angiotensiongen, angiotensin converting enzyme, AT1 receptor, VEGF, HIF 1 alpha and HPRT (housekeeping gene) expression were analyzed using Real Time PCR (BioRad C1000 Thermal Cycler). mRNA was extracted using mRNA extraction column based kit (RNeasy kit, invirogen). cDNA was synthesized and qPCR was performed according to the manufacturer protocol (Reverse aid cDNA kit, Thermo scientific). 50ng of cDNA was used for PCR amplification.

The forward and reverse primer sequences of RAS components, VEGF & HIF-1α are as following-

**Renin**
(NM_012642.4, F-CCTTTGGAGAGGTCACCGAG, R -GACTCCATCAACAGCCTGAGCAG)

**Angiotensinogen**
(M12112.1, F-CACTCCTTCCAGCAA GGC AG, R-TCT CAC CCC AGT GTC CAA AC)

**Angiotensin Converting Enzyme**
(NM_012544.1, F-GTGGGTATCCCACTGAAGCC, R-CATACCAGGAGCGCCAGTAG)

**AT1 receptor**
(NM_031009.2, F-GTTTGCCAGCCGTCATCTAC,R-GTCCAATGGGGAGTGTTGAG)

**VEGF**
(NM_001287114.1, F-TCATCAGCCAGGGAGTCTGT, R-GCAACCTCTCCAAACCGTTG)

**HIF 1 Alpha**
(NM_024359, F-AAGTCTAGGGATGCAGCACG, R-TAGATGGGAGCTCACGTTGTG)

**HPRT**
(NM_012583, F-CCCCAAAATGGTTAAGGTTGC, R-AACAAAGTCTGGCCTGTATCC)

Reactions were performed with the following thermal profile of 40 cycles of 95°C for 15 s, 58°C for 20 s and 72°C for 15 s.

The normalized gene expression level of renin, angiotensinogen, angiotensin converting enzyme, AT1 receptor, VEGF and HIF 1 alpha was derived by using the ΔΔCt method as the difference of ΔCt between target and reference gene (HPRT). Real time experiments were performed in triplicate for each gene including HPRT. The threshold cycle at which the increase in the signal with exponential growth of PCR products was detected was obtained for the quantification.

#### Quantification of lisinopril and telmisartan using LC-MS/MS

Lisinopril and telmisartan in the vitreous and plasma samples (n = 9) were estimated using liquid chromatography (Surveyor, Thermo, USA) coupled tandem mass spectroscopy (4000QTrap, Absciex, USA). Briefly, the analytical separation of both the compounds was achieved using a phenyl-hexyl column (Merck, Germany) with the gradient elution with water (with 0.1% formic acid) and methanol (Merck, Germany) at flow rate of 200μl/min to elute both analytes simultaneously. Sulfadimethoxine (SDM) was used as the internal standard. For the quantification multiple reaction monitoring mode was used with the transitions of 406.1/246.1, 309.5, 515.2/276.2, 305 and 311/129 for lisinopril, telmisartan and sulfadimethoxine respectively. All other source and compound dependent parameters were optimized using the inbuilt algorithm to get maximum ions intensity in the analysis to reach required sensitivity.

On the day of analysis, ocular tissues and plasma of the pups were thawed, deproteinated and extracted for quantification using the aforesaid method. Briefly, 5μg of tissues or 5μl of plasma/vitreous were added with 100μl of extraction solvent (95% methanol having 50ng/ml internal standard) and subjected for vortexing and centrifugation at 8800g for 10min. The supernatant was transferred into 96well plates and 5μl was injected for the quantification. Appropriate spiked standards were used for the construction of calibration curve for the calculation of unknown concentration using inbuilt algorithm of Analyst Ver. 1.5.2.

Descriptive statistics to determine mean, standard deviation and standard error of mean was performed with appropriate statistical tests using Sigma Stat software (Version 3.5). ‘p’ value was taken two tailed and a value <0.05 was considered to be statistically significant. Inter-group analysis was done using the unpaired t- test and intra-group analysis was done using Paired t- test.

REST (Relative Expression Software Tool) software was used for the statistical analysis of the gene expression studies and a value <0.05 was considered to be statistically significant [13].

## Results

### Human Study

#### Demographic profile of patients

**Fig 1:**
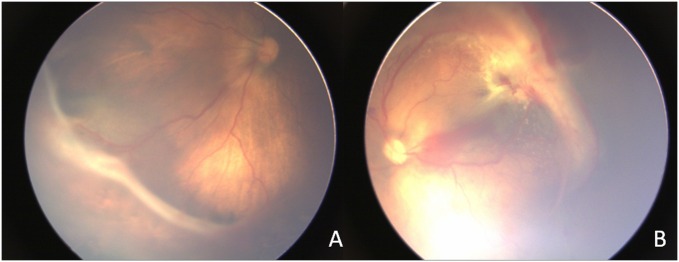
Representative fundus images. Fig 1 shows the fundus images of human subjects with stage 4A (A) and 4B (B) ROP. The stages were classified according to ICROP (International classification of Retinopathy of Prematurity).

Out of 44 patients enrolled in the study, 44% were female and 56% were male. 61.36% of the patients were having stage 4A ROP, whereas 38.63% of the patients were suffering from 4B ROP. The study population was from northern part of India with the South Asian ethnicity. The stage of ROP was diagnosed and classified by the experienced ophthalmologist after thorough fundus examination (Fig 1). Birth weight of ROP population ranged from 900–1850gm. The majority of study population (n = 44) was between the age group of 0–6 months (79.54%) followed by 06–12 months (13.63%), 12–36 months (4.54%) and 36–72 months (2.27%). The control group (n = 12) had 38% of female and 62% of male, and 62.5% patients were in 0–6 months, 12.5% in 6–12 months and rest 25% were in 12–18 months group.

#### Total protein concentration

**Fig 2:**
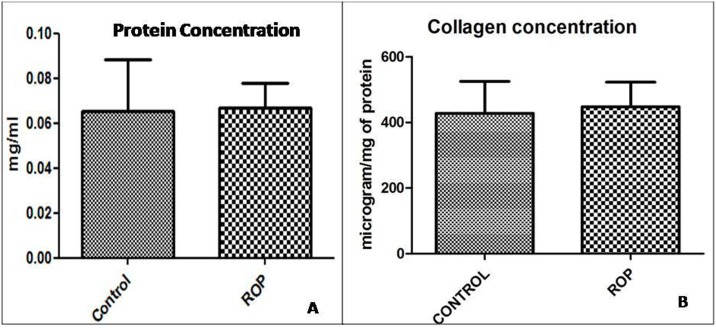
Protein and Collagen conc. Vitreous obtained from the human ROP and Control subjects were evaluated for the presence of the (A) total protein and (B) total collagen. No significant differences were observed between the groups. Data is presented as mean ±SEM.

The total protein concentration found in the vitreous of control and ROP were 0.065± 0.023 and 0.067± 0.006 mg/ml respectively. No statistical difference was found in the protein concentration of both groups (Fig 2A).

#### Total collagen concentration

The total collagen concentration found in the vitreous of control and ROP were 427.9 ± 97.48 and 447.5 ±75.14 μg/mg of protein respectively. No statistical difference was found in the total collagen concentration of both groups (Fig 2B).

#### ELISA for RAS component, VEGF and HIF 1 alpha

**Fig 3:**
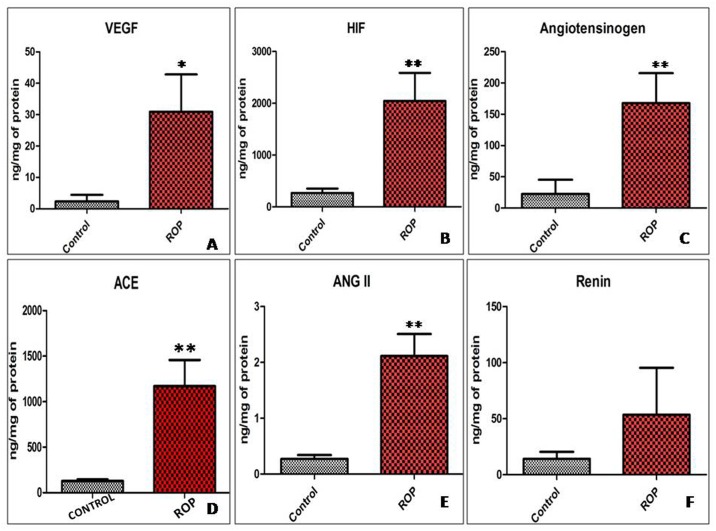
Protein concentration of RAS components and angiogenic factors. Human vitreous of disease (n = 44) and control patients (n = 12) were subjected for the quantification of (A) VEGF- vascular endothelial growth factor, (B) HIF1α-Hypoxia inducible factor 1 alpha, (C) angiotensinogen, (D) ACE-Angiotensin Converting Enzyme, (E) ANG II- angiotensin II and (F) Renin. All concentrations were normalized as per mg/protein. Data is represented as mean ±SEM, significant difference found in comparison with respective control (***p≤0.001, **p≤0.01, *p≤0.05), using unpaired student t-test.

A statistically significant fold increase of 12, 7, 6.3, 9.5 and 8 was observed in the levels of VEGF, HIF 1 alpha, angiotensinogen, angiotensin converting enzyme and angiotensin II in the disease group as compared to control. Though the renin concentration in vitreous of ROP patients was found to be 5 fold increased as compared to control, this was not statistically significant (Fig 3).

### Animal Study

#### ROP rat model study

A prospective randomized study was carried out on neonatal pups (n = 9 each group). The pups were weighed at 7 day and day 17. The weights were compared to the room- air raised rats. The average body weight of rat pups at day 7 was 8 gm (±1.5 gm), at day 17 the avg. body weight of normal rat pups was 18.9 gm (±1.7 gm), whereas the avg. weight of hyperoxia treated group was 16 gm (±1.2 gm) which was lesser than the normal but not significant. Upon treatment with various drugs the avg. weight of lisinopril, telmisartan and bevacizumab treated groups at postnatal day 17 were 16.5 ± 1, 18.8 ± 1.5 and 17.3 ± 0.3 respectively, whereas the avg. weight of disease control and normoxia pups at day 17 were 18.9 ±1.5 and 24.6± 0.21 respectively. The decrease in body weight was observed in all treatment groups, as well as in disease control group as compared to room air raised animals, though the difference was not statistically significant.

#### Analysis of retina using fundus imaging

**Fig 4:**
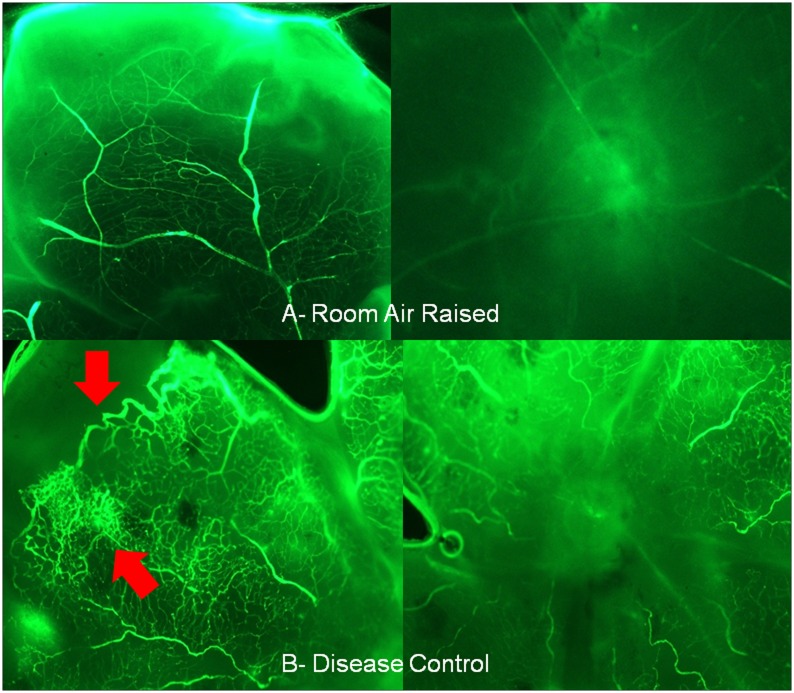
FITC conjugated dextran retinal flat mount. Arrows show the avascularised junction between the mid zone of retina and the ora serrata along with microaneurysm using FITC dextran labelling (A) Normal group- fully vascularised retina (B) Hyperoxia group- avascularised zone can be seen towards peripheral retina.

The establishment of OIR model was assessed using the FITC dextran labeling. Fig 4 shows the ischemic damage to the OIR model retina. After disease induction rat pups were treated with test drugs and retina was evaluated by means of fundus imaging using MICRON-III retinal imaging system on day 17. As mentioned in methods, tortousity index (TI) was calculated for retinal vessels.

On comparing the tortousity index of arteries (TI_A_) and veins (TI_V_), it was noted that the TI_A_ was much higher than TI_V_, thus showing a greater effect of the disease process on arteries than veins in the rat model of ROP. When compared to room air raised rats, the TI_A_ in disease control was 31% higher than room air (TI_A_ = 1.029) which was statistically significant (p = 0.002), hence the disease model of ROP was reliably produced in our laboratory (5A).

**Fig 5:**
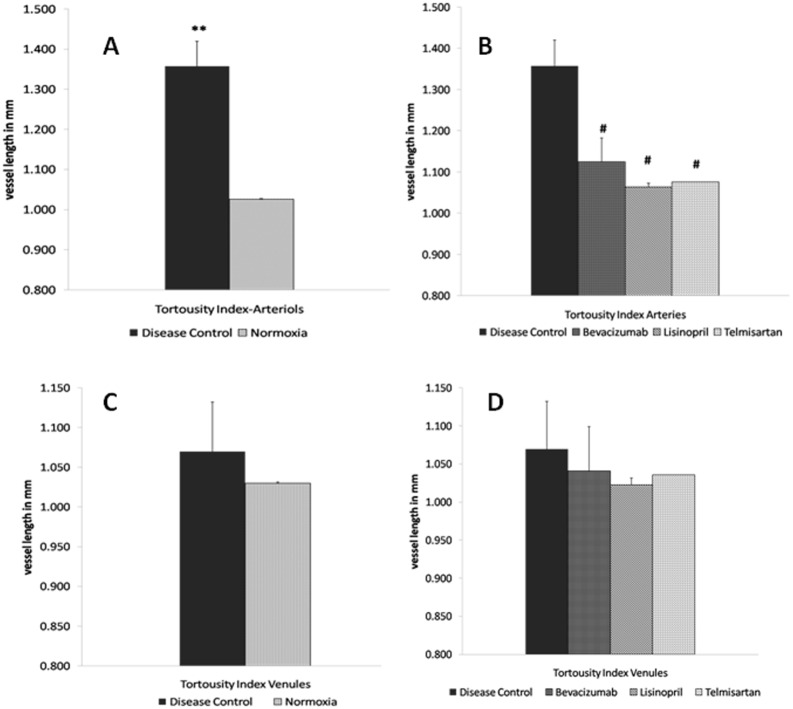
Tortousity Index. Fig 4A is showing the significant increase in tortousity index of disease control as compared to normoxia (p≤0.01), whereas fig 4B is depicting the significant fall in the TI of treatment groups in comparison with disease control. Fig 4C & 4D tortousity index of veins in various groups, where no significant difference was observed. Data is represented as mean ±SEM, significant difference found in comparison with respective control (***p≤0.001, **p≤0.01, *p≤0.05), using unpaired student t-test (n = 9).

The tortousity of vessels at the posterior pole was higher in all the groups kept on hyperoxia as compared to room air. The tortousity index of disease control was higher (TI_A_ = 1.349), followed by bevacizumab (TI_A_ = 1.167), lisinopril (TI_A_ = 1.02) and telmisartan (TI_A_ = 1.0794) treated groups. The tortousity index in bevacizumab, lisinopril (p ≤ 0.018) and telmisartan (p = 0.002) treated groups, significantly reduced when compared to disease control group (Fig 5B).

The tortousity index of disease control veins (TI_V_ = 1.070) were found to be increased when compared to normoxia (TI_V_ = 1.030) rat pups, although not significantly (Fig 5C). When various treatment groups i.e. bevacizumab (TI_V_ = 1.041), lisinopril (TI_V_ = 1.023) and telmisartan (TI_V_ = 1.018) veins tortousity index were compared to disease control, no statistical significance was found although there was decrease in all treatment groups (Fig 5D).

#### Functional analysis of retina using ERG

**Fig 6:**
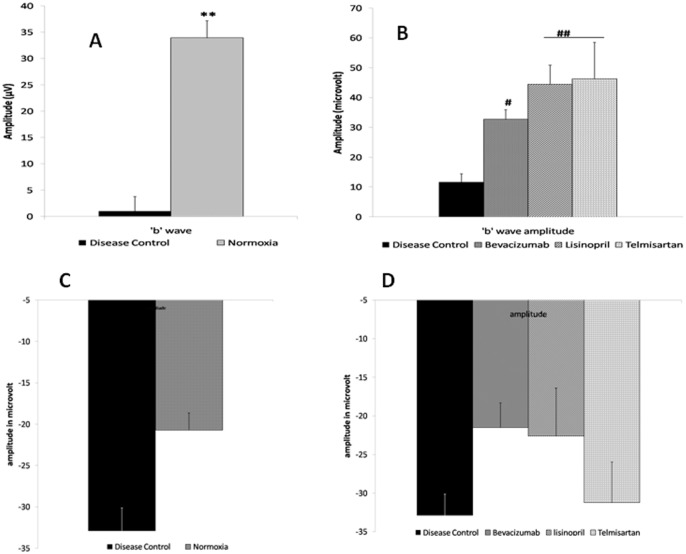
ERG ‘b’ & ‘a’ wave. ‘b’ wave significantly decreased (*p≤0.01) in disease control group (6A). Fig 6B depicting the improved ‘b’ wave response in all treatment group in comparison with disease control. Fig 6C & 6D showing the ‘a’ wave response of various groups. Data is represented as mean ±SEM, significant difference found in comparison with respective control (***p≤0.001, **p≤0.01, *p≤0.05), using mann-whitney test (n = 9).

Electroretinographic evaluation revealed that ‘b’ wave amplitude of disease control (11.62 μV) was reduced significantly (p = 0.002) when compared to that of room air raised rat pups (33.96 μV) on postnatal day 17 (Fig 6A). The treatment with test drugs i.e. lisinopril (44.45 μV), telmisartan (40.344 μV) and positive control bevacizumab (32.71 μV) was able to restore the functional health of retina and significantly improved the ‘b’ wave response in the respective groups (Fig 6B). The amplitude of ‘a’ was slightly elevated in disease control group as compared to room air raised (Fig 6C) and no significant changes were observed in amplitude of ‘a’ wave in treatment groups in comparison to disease control (Fig 6D).

**Fig 7:**
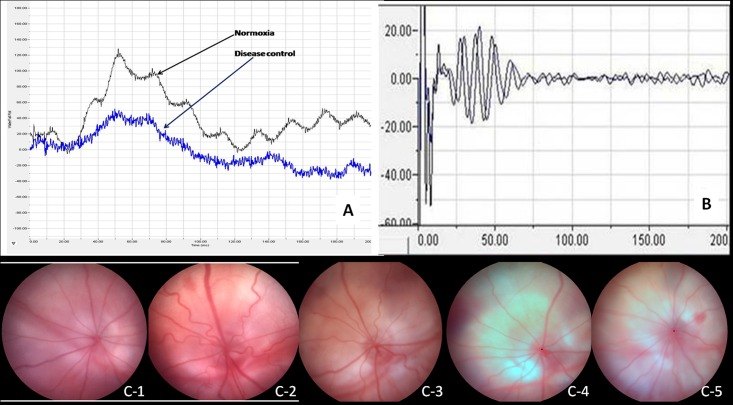
ERG, OPs & Fundus images. Representative graphs for (A) electroretinogram, (B) oscillatory potential. Whereas (C1–6) represents (C1) disease control, (C2) normoxia, (C3) bevacizumab, (C4) lisinopril, (C5) telmisartan group fundus images.

ERG was also evaluated through the mean of latency of ‘a’ and ‘b’ wave. The latencies for ‘a’ wave of different groups were 23.18 msec, 26.43 msec, 23.08 msec, 22.22 msec, and 28.50 msec for normoxia, disease control, bevacizumab, lisinopril and telmisartan respectively. The latency of ‘b’ wave for normoxia, disease control, bevacizumab, lisinopril and telmisartan were 78.88 msec, 82.08 msec, 77.32 msec, 52.32 msec and 68.23 msec respectively. No significant difference was found in the latencies of ‘a’ and ‘b’ wave of various groups. The representative ERG graphs are shown in Fig 7A.

#### Oscillatory potentials

The oscillatory potentials (OPs) were filtered using narrowing down the bandwidth, representative oscillatory potentials are shown in Fig 7B. Representative fundus images for disease control, normoxia and all treatment groups are shown in Fig 7C.

**Fig 8:**
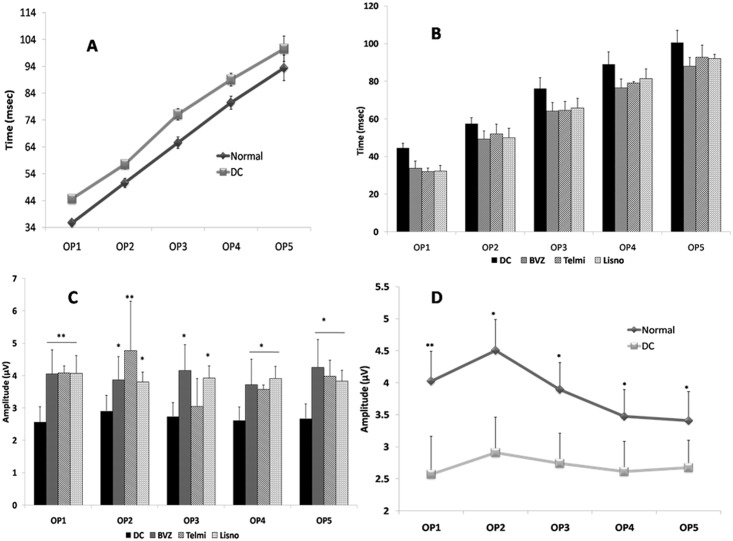
Oscillatory potential amplitude & latency. Fig 8A represents Oscillatory potentials (OPs) timing of disease control (DC) and normal and 8B represents the timings of OPs of various treatment groups with comparison to DC. Fig 8D shows the significant elevation of OPs amplitude in normal as compared to DC and fig 8C represents the significantly increased amplitude of test groups in comparison with DC. Data is represented as mean ±SEM, significant difference found in comparison with respective control (***p≤0.001, **p≤0.01, *p≤0.05), using mann-whitney test (n = 9).

Initial 5 oscillatory potentials originated from the neonate retina were analyzed for the amplitude and the latency. The oscillatory potentials of disease control retina was delayed by 8.41 msec when compared with the normal group retina, and as far as amplitude was concerned, a significant decrease of all the oscillatory wavelets were observed (p≤0.002) in disease control group. The latency delay of OPs was recovered in all the treatment groups and significant increase in amplitude of OPs was observed in telmisartan, lisinopril and bevacizumab group, when compared with the disease control group (Fig 8).

#### Gene expression studies on RAS components and angiogenesis factors

RAS components (renin, angiotensinogen, angiotensin converting enzyme (ACE), AT1 receptor), VEGF and HIF-1α were analyzed through qPCR.

**Fig 9:**
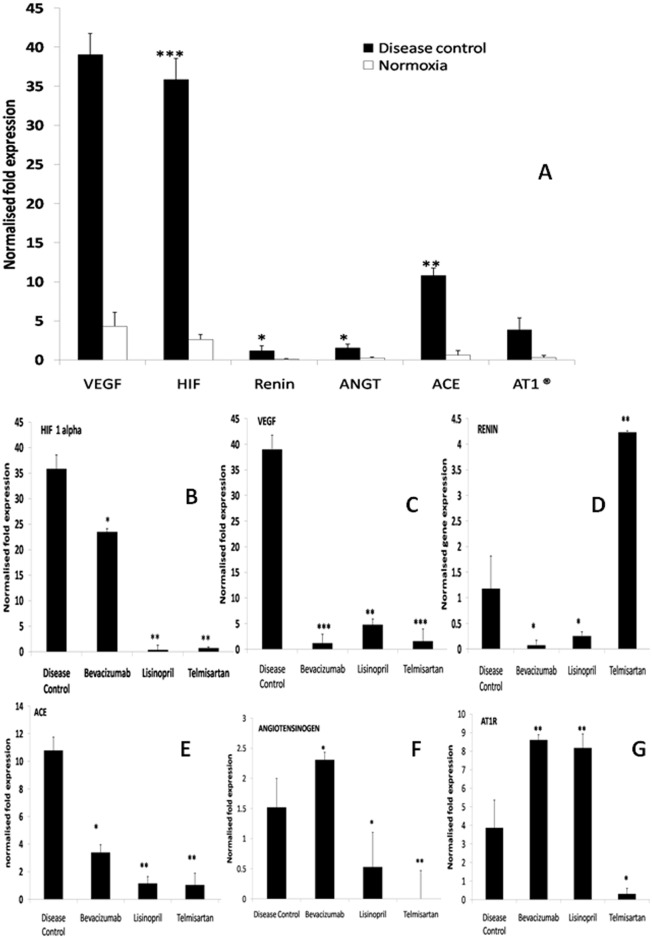
Gene expression analysis. Fig 9A shows the significant multiple folds up regulation of VEGF- vascular endothelial growth factor, HIF1α- Hypoxia inducible factor 1 alpha, angiotensinogen, ACE-Angiotensin Converting Enzyme, AT1 receptor and renin in disease control group in comparison to normal group. Fig 9B, 9C, 9D, 9E, 9F and 9G is representing the normalised expression of HIF 1 α, VEGF, renin, ACE, angiotensinogen and AT1 receptor in various test groups in comparison to disease control. Data is represented as mean ±SEM, significant difference found in comparison with respective control (***p≤0.001, **p≤0.01, *p≤0.05), using Rest (relative expression software tool). DC-disease control, BVZ-bevacizumab, LIS-lisinopril, TELM-telmisartan (n = 9).

When expression of all genes in room air raised group were compared with the hyperoxia treated group, a significant increase was observed in VEGF, HIF 1α, renin, angiotensinogen, ACE, AT1 receptor (Fig 9A).

The expression of HIF-1α was 34 fold up-regulated significantly in disease control retina (p≤0.001), when compared to retina of room air raised pups. In lisinopril and telmisartan group a significant down regulation was observed for HIF-1α (p≤0.01) which was 36 and 35 fold less respectively in comparison to disease control. There was also significant decrease in expression of HIF-1α in the bevacizumab group (p≤0.05) in comparison to disease control (Fig 9B).

Vascular endothelial growth factor (VEGF) expression was significantly raised (p≤0.001) to 35 fold when compared to normoxia rat pups at PN 17. The expression of VEGF reduced significantly in all the treatment groups. The expression of VEGF was down regulated in bevacizumab 38 fold (p≤0.001), lisinopril 34.30 fold (p≤0.01) and telmisartan 37.497 fold (p≤0.001) when compared with the expression in disease control retina (Fig 9C).

The expression of renin in disease control retina was found to be increased by 1.091 fold (p≤0.05), when compared to the expression in room air raised rat pups. There was also significant down regulation of its expression by 1.10 fold in bevacizumab (p≤0.05), 0.93 fold in lisinopril (p≤0.05) and up regulation in telmisartan group (p≤0.01) which was increased by 3.05 fold in comparison to disease control (Fig 9D).

The expression of ACE was significantly up regulated by 10.18 fold in disease control group (p≤0.01) from room air raised group. The expression of ACE was significantly reduced in treatment group’s retina also when compared with disease control. The expression of ACE down regulated by 7.41 fold in bevacizumab (p≤0.05), 9.66 fold in lisinopril (p≤0.01) and 9.75 fold in telmisartan (p≤0.01) when compared to disease control (Fig 9E).

Angiotensinogen expression was 1.24 fold up regulated in the disease control group as compared to normoxia rat pups; the increase was statistically significant (p≤0.05). The expression of angiotensinogen was significantly reduced to 0.98 fold in lisinopril group (p≤0.05) and to 1.51 fold in telmisartan group (p≤0.01) in comparison to disease control, but significantly increased expression was observed in bevacizumab (p≤0.05) by 0.79 fold when compared to disease control group (Fig 9F).

AT1 receptor expression was 3.56 fold increased (p≤0.05) in disease control group in comparison with room air raised group. AT1 expression was found to be significantly up regulated in lisinopril (p≤0.01) and bevacizumab (p≤0.01) group by 4.30 and 4.73 fold respectively. A significant down regulation in expression of telmisartan (p≤0.05) was observed of 3.55 fold (Fig 9G).

#### Levels of lisinopril and telmisartan in plasma and ocular tissues

**Fig 10:**
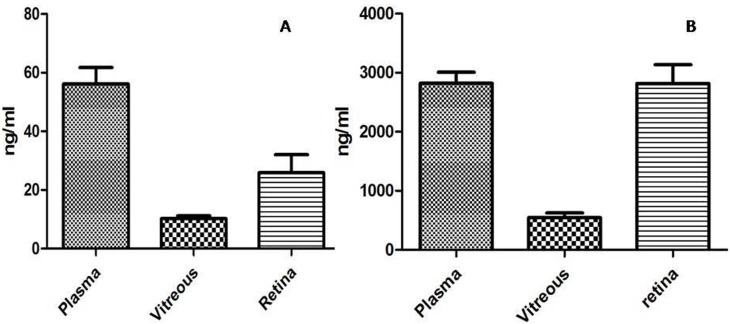
Levels of Lisinopril & Telmisartan. Fig 10A shows the telmisartan levels and fig 10B shows the lisinopril levels in the plasma, vitreous and retina. Data is represented as mean ±SEM, significant difference found in comparison with respective control (***p≤0.001, **p≤0.01, *p≤0.05), using unpaired student t-test (n = 9).

Telmisartan showed 56.2± 5.6, 10.23± 0.9 ng/ml and 25.92± 25.9 ng/ml in plasma, vitreous and retina respectively (Fig 10A). Plasma, vitreous and retinal levels of lisinopril was found to be 2821 ± 188, 545.1 ± 80.2 ng/ml and 2119.6 ± 314.9 ng/ml respectively (Fig 10B).

## Discussion

Retinopathy of Prematurity (ROP) is a potentially blinding eye disorder that primarily affects premature infants born before 32 weeks of gestation having low birth weight of 1.5 Kg or less. Intravitreal pegaptanib or bevacizumab injection alone or in combination with laser photocoagulation are so far considered as therapeutic modalities for the management of ROP [14] but the systemic toxicity of intravitreal bevacizumab is of concern [15].

Hypoxia induced activation of RAS has been reported in various tissues [16]. These tissue specific RAS are speculated to be involved in the pathophysiology of tissue/organ functions. Retina is known to have mechanisms for the production of erythropoietin [17] and renin [1]; therefore, their role in retinal hypoxic conditions has been felt as important in retinal neovascularization and its related pathological manifestations.

RAS has been reported to be found in blood vessels and ganglion cell layer which has been implicated in the early stages of vascularization in the animal models [9]. Gene expression studies have identified that a tissue-based RAS (intrinsic pathway) exists in the adult eye. As RAS presence and activation has been found in the animal models and in human eyes, the present study evaluated the cascade of events involved in the pathophysiology of ROP to delineate the role of pharmacological intervention in reducing or arresting the progression of the changes and to preserve the retinal function in ROP.

In order to understand the possible involvement of RAS in ROP, this study quantified the vitreous levels of RAS components and angiogenic cytokines in the vitreous of patients undergoing vitrectomy surgery for ROP and compared with their age matched control where vitrectomy is warranted for surgical management of congenital cataract. In this study majority of infant population was less than 6 months of age. Vitreous samples were acquired from the first intervention at our centre for the surgical management of leaky aberrant vessels in the infants of ROP. Moreover, the subjects were recruited only from the populations who have not undergone any anti-VEGF therapy before surgical intervention. Thus, the RAS components and angiogenic cytokines levels were not influenced by any past treatment to represent underlying disease pathology.

In this study a significant increase was observed in the RRAS components and angiogenic cytokines in the vitreous humour of infants with ROP as compared to their respective age matched control group.

A several fold rise was observed in the levels of RAS components such as renin (5 folds), angiotensinogen (6 folds), ACE (10 folds), ANG II (8 folds) and angiogenic cytokines such as VEGF (12 folds) and HIF-1α (7 folds) in the vitreous humor of infants with ROP as compared to the vitreous of infants operated for congenital cataract. Interestingly, this study found 5 fold increase in renin levels in ROP group as compared to control but the increase was found to be statistically insignificant, indicating the possibility of up regulation of angiotensinogen which could be a key mechanism for the enhancement of downstream pathway leading to production of vaso-active component such as ANG-II (8 folds). From this analysis it is evident that the multiple fold increase in RAS components has a major role in the pathophysiology of this condition. In order to explore its therapeutic role in the condition of ROP, animal studies were performed and the role of RAS was evaluated using pharmacological interventions.

The results of this study showed multiple fold up-regulations of the mRNA expressions for renin, angiotensinogen, ACE & AT1 receptor in the retina of OIR pups as compared to pups kept in normoxia. The up-regulation of renin, angiotensinogen, ACE and AT1 receptors are clearly indicating the predominant role played by the RRAS system in the pathophysiology of OIR. Both ACEI and AT1 receptor blockers significantly attenuated the expression of VEGF, indicating that RRAS is playing a key role in the vascular proliferation pathway and could be a potential modality for the treatment of ROP. It can be further supported by the findings from transgenic Ren-2 rats that ACE inhibition attenuated hypervascularization of the developing retina [9].

OIR model is well accepted model to study retinal angiogenesis in ischemic retinopathies. FITC dextran has been used to map the angiogenesis in OIR model in the retinal flat mounts. While adopting the similar procedure, the present study has also observed an avascularised junction between the mid zone of retina and the ora serrata along with microaneurysm which are similar to the findings of others [18, 19, 20]. In order to compare the effect of RRAS blockade with control, this study opted for ERG and retinal vascular tortousity as quantifiable parameters.

The results of ERG studies revealed a significant fall in ‘b’ wave amplitude as compared to the pups of normoxia group, where as ‘a’ wave remained unaltered. This indicates that functionally inner retinal layers are affected by OIR on day 17 in this model. Moreover, tortousity index of the OIR pups was significantly increased as compared to normoxia group which were raised in room air indicating the similarities with human plus (+) disease of ROP. Progressive vascular incompetence occurring along with the changes described at the edge of the abnormally developing retinal vasculature is noted as increasing dilatation and tortousity of the peripheral retinal vessels, in this condition a plus sign is added to the ROP stage number and called as plus disease [21]. Similar observations of decreased b-wave amplitude and increase in tortousity index in the model of OIR has been reported by Favazza et al (2013) [22].

Beside, ‘a’ and ‘b’ wave, Oscillatory Potentials (OPs) are the sensitive markers (OP 1–5) hidden in the ascending and descending arm of ‘b’ wave of ERG. These markers reflect the alteration in retinal layer communication in a variety of disease such as retinopathies and glaucoma [23]. In ROP rats, marked attenuation of OPs are consistent with persistent dysfunction indicating the compromised function of inner retinal circuitry [24]. In OIR model, amplitude was significantly reduced as compared to normoxia group and was successfully restored upon treatment with bevacizumab, ACEI and ARB.

In order to evaluate the effect of ARB in this study, telmisartan has been included considering its longer half life and higher affinity for AT1 receptor. Nakamura and coworkers (2009) used a pro-drug olmesartan medoxomil orally to the pups which was expected to be converted to its active form in the intestine [25]. As the bioavailability of the active drug in a neonate is an unreliable factor, the present study used telmisartan. Similarly, lisinopril was used as an ACEI due to its active form and doesn’t require any metabolic transformation for its activity. These drugs were injected through intradermal route 48 hours apart to avoid drug related toxicity. To block the end of the cascade, VEGF antibody (bevacizumab) was selected and considered as a positive control.

Both lisinopril (ACEI) and telmisartan (ARB) significantly blocked the up regulation of VEGF and HIF-1α successfully indicating that RAS activation is the primary cascade involved in evoking changes in retinal functions and vasculature morphology. On the day P17, retinal function analysis revealed that fall in ‘b’ wave amplitude in control (untreated) animals was restored completely by the inhibition of RAS cascade either by ACEI or ARB. VEGF antibody (Bevacizumab) therapy had also attenuated the expression of VEGF and renin but was unable to neutralize the expression of other RAS components indicating that anti-VEGF therapy alone may not be able to abolish the RAS cascade for a prolonged duration. Moreover, arteriolar vessel tortousity was also not completely attenuated by bevacizumab indicating that it may not be able to stop other factors involved in the HIF mediated pathway. Angiotensin II is considered as the most active component of the RAS, it is reported to be a potent HIF-1α activator in vascular smooth muscle cells and is involved in its transcription, translation, and protein stabilization [26].

In renin over expressing transgenic (mRen-2) rat model, lisinopril reduced both retinal vascular endothelial growth factor and its type 2 receptor mRNA in ROP rats, whereas losartan (ARB) had no effect [27]. In this study we have assessed drug levels in the vitreous and retina on PN17 in the pups. After intradermal administration, retina to plasma ratio for lisinopril and telmisartan were found to be 0.99 and 0.46 respectively. This data reveals that both lisinopril (IC_50_ 0.1nM) and telmisartan (IC_50_ 0.49nM) could reach adequate levels in the retina above their IC_50_ values indicating that the observed effect is due to the penetration of these agents.

## Conclusion

For the first time this study clearly showed the involvement of RAS components in ROP and in developing retina of infants. The significant increase of the RAS components in the vitreous is the proof that this system might be playing a major role in ROP and indicating the possibility of exploring the key targets such as ACE and angiotensin receptors for pharmacological interventions. Subsequent experimental studies in animals confirmed the involvement of RRAS components in OIR rats. Blockade of either ACE or angiotensin receptor in rat pups suppressed the over-activation induced vascular and retinal functional changes in ROP.
